# Multi-omics Mendelian randomization integrating RNA-seq, eQTL and pQTL data revealed CPXM1 as a potential drug target for osteoporosis

**DOI:** 10.1186/s41065-025-00562-w

**Published:** 2025-09-30

**Authors:** Junxiang Lian, Xinjian Zhang, Shanwei Shi, Xinping Li, Zhiping Wang, Hailing Pang, Tuo Wang, Wenfeng Gao, Xinpeng Liu

**Affiliations:** 1 Department of Oral and Maxillofacial Surgery, Stomatological Hospital, School of Stomatology, Southern Medical University, Guangzhou, 510280 China; 2 Jiangxi Provincial Key Laboratory of Oral Diseases, Department of Stomatology, The First Affiliated Hospital, Jiangxi Medical College, Nanchang University, Jiangxi 330006 Nanchang, China; 3 Department of stomatology, The people’s hospital of laibin, Laibin, 546100 Guangxi China

## Abstract

Osteoporosis, a prevalent skeletal disorder characterized by decreased bone mineral density and increased fracture risk, continues to be a major global health concern. Traditional treatments for osteoporosis have limited efficacy and safety profiles, highlighting the need for novel therapeutic targets. This study integrates multi-omics data, including RNA-seq, expression quantitative trait loci (eQTL), and protein quantitative trait loci (pQTL) data, through Mendelian randomization (MR) to identify potential drug targets for osteoporosis. By leveraging bidirectional two-sample MR analysis, we identified CPXM1 (Carboxypeptidase X, M14 family member 1) as a novel gene that is causally linked to osteoporosis risk. Through transcriptomic and proteomic validation, we demonstrate that CPXM1 was upregulated in aged bone tissues and osteoporotic conditions in both human and murine models. Gene set enrichment analysis (GSEA) revealed significant dysregulation of bone homeostasis pathways, including increased extracellular matrix degradation and suppression of osteoblast differentiation in aged mice. Furthermore, phenome-wide association studies (PheWAS) confirmed minimal off-target effects of CPXM1, reinforcing its potential as a therapeutic target. Finally, computational drug repurposing predicted several promising drug candidates, including Doxorubicin, 5-Fluorouracil, and 2-Methylcholine, which may target CPXM1 pathways for osteoporosis treatment. These findings highlight CPXM1 as a potential biomarker and therapeutic target, offering new avenues for osteoporosis therapy.

**Supplementary Information:**

The online version contains supplementary material available at 10.1186/s41065-025-00562-w.

## Introduction

Osteoporosis, a systemic skeletal disorder characterized by reduced bone mineral density (BMD) and increased fracture risk, imposes a significant public health burden globally, particularly among the aging population [1]. Traditional therapeutic modalities, such as bisphosphonates and selective estrogen receptor modulators, are often limited by suboptimal efficacy and potential safety concerns, underscoring the imperative need for innovative therapeutic strategies [2, 3]. The identification of novel drug targets remains a formidable challenge, given the multifaceted etiology of osteoporosis, which encompasses intricate interplay among genetic, molecular, and environmental factors. However, the advent of multi-omics technologies, encompassing transcriptomics, proteomics, and genomics, has revolutionized our understanding of osteoporosis by providing a comprehensive and integrative approach to elucidate its molecular underpinnings and identify potential therapeutic targets [4].

Mendelian randomization (MR) analysis, leveraging genetic variants as instrumental variables, has emerged as a robust method to infer causal relationships between exposures and outcomes, thereby identifying potential drug targets [5]. Recent methodological advancements in multi-omics MR frameworks enable simultaneous interrogation of transcriptomic, proteomic, and genomic datasets, offering unprecedented opportunities to disentangle molecular causality networks in complex disorders [6]. While genome-wide association studies (GWAS) have identified over 500 loci associated with BMD variation [7], the functional interpretation of these signals and their translation into therapeutic targets remain elusive, primarily due to incomplete understanding of gene regulatory mechanisms and protein-disease pathways.

The integration of expression quantitative trait loci (eQTLs) and protein quantitative trait loci (pQTLs) with disease-associated variants addresses this critical gap by establishing mechanistic links between genetic determinants and their molecular effectors [8]. Our study pioneers the application of tri-omics MR (RNA-seq, eQTL, and pQTL) in osteoporosis research, systematically prioritizing targets through concordant evidence across multi-layered molecular datasets. This causal inference paradigm leverages bidirectional two-sample MR to dissect genetically proxied biomarker-disease relationships while implementing reverse causality analysis to identify off-target pharmacological effects – a methodological innovation that simultaneously advances target discovery and preclinical safety profiling.

## Methods

### Study design

**Fig. 1:**
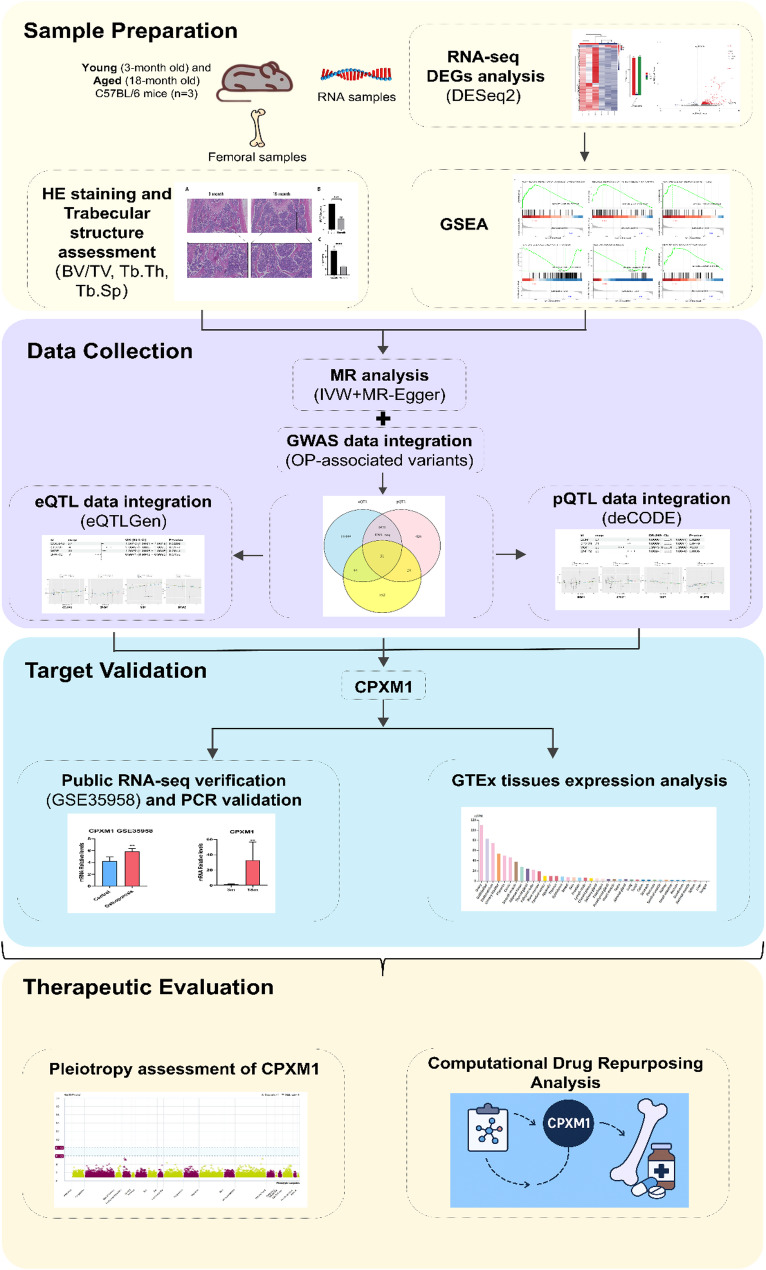
Workflow of the study design

This study design was shown as Fig. 1.

### Histological analysis via hematoxylin-eosin (HE) staining

Femoral bones were harvested from 3-month-old (young, *n* = 3) and 18-month-old (aged, *n* = 3) C57BL/6 mice under sterile conditions. Fresh specimens were fixed in 4% paraformaldehyde (PFA) at 4 °C for 48 h, then decalcified in 14% EDTA (pH 7.4) with gentle agitation for 21 days. Decalcification was verified by radiographic imaging. Coronal bone sections were mounted on poly-L-lysine-coated slides and subjected to hematoxylin-eosin (HE) staining. Nuclei were stained with hematoxylin, differentiated in 1% ethanol, turned blue in 0.2% ammonia solution, and cytoplasm was counterstained with eosin Y. Sections were then dehydrated and mounted using neutral resin.

Histological evaluation of trabecular architecture in the distal femoral metaphysis (0.5–1.5 mm proximal to the growth plate) was performed under 20× magnification using a Nikon microscope. Bone volume fraction (BV/TV) and bone surface (BS) were assessed qualitatively based on visual scoring criteria by three independent blinded observers. Scoring criteria were adapted from previously published semi-quantitative histological assessment methods [9, 10].

BV/TV was scored on a 5-point scale:

1 = almost no trabeculae,

2 = sparse trabeculae,

3 = moderate density,

4 = dense trabeculae,

5 = very dense, tightly packed trabeculae.

BS was evaluated using a separate 5-point scale:

1 = smooth, minimal surface,

2 = limited surface undulation,

3 = moderate surface area and contour,

4 = increased surface complexity,

5 = highly irregular and extensive bone surface.

Average scores from both observers were used for statistical analysis. Data were analyzed using a two-tailed Student’s t-test in GraphPad Prism v9.0, with statistical significance set at *P* < 0.05.

### Analysis of RNA sequencing

Three 3-month-old and three 18-month-old C57BL/6 mice were euthanized under strict aseptic conditions for bilateral femoral bone collection. Following femoral artery ligation, soft tissue dissection was performed to isolate intact femurs, which were subsequently stripped of periosteum and residual musculature using microdissection forceps. Fresh bone tissue specimens were harvested within a 2-minute window to minimize RNA degradation, followed by three sequential PBS washes to remove extraneous connective tissues, adipose deposits, and surface contaminants. Processed samples were immediately transferred to RNase/DNase-free tubes and flash-frozen in liquid nitrogen for 30 min to preserve RNA integrity. Total RNA was isolated from bone tissue using TRIzol^®^ Reagent followed by rigorous quality control: absorbance ratios (A260/A280) were quantified via Nanodrop ND-2000 (Thermo Scientific), and RNA integrity (RIN > 8.0) was verified using an Agilent Bioanalyzer 4150. Sequencing was performed by Applied Protein Technology Co., Ltd. (Shanghai, China) using the Illumina Novaseq 6000 / MGISEQ-T7. Qualified RNA underwent library preparation with the ABclonal mRNA-seq Kit, involving mRNA capture by oligo(dT) beads, thermal fragmentation, double-stranded cDNA synthesis (reverse transcriptase/DNA polymerase I), adapter ligation, and PCR amplification. Purified libraries (AMPure XP) were validated on the Bioanalyzer. Differential expression analysis was performed using the DESeq2 (http://bioconductor.org/packages/release/bioc/html/DESeq2.html), differentially expressed genes (DEGs) with |log2FC|>0.5 and adjust *P* value < 0.05 were considered to be significantly different expressed genes. We have deposited the raw sequencing data in the Sequence Read Archive (SRA) under accession number PRJNA 1,305,700.

### Gene set enrichment analysis (GSEA)

Functional enrichment analyses, including Gene Ontology (GO) terms, Kyoto Encyclopedia of Genes and Genomes (KEGG) pathways, and gene set enrichment analysis (GSEA), were conducted using the Dr. Tom platform (https://bio.tools/dr_tom). Gene sets were compiled from the Molecular Signatures Database (MSigDB v7.5) [11], encompassing canonical pathways, biological processes, and osteoporosis-related signatures. RNA-seq expression profiles were pre-ranked by log2 fold change values, and enrichment significance was evaluated using 1,000 permutations. Gene sets meeting the following thresholds were considered significantly enriched: normalized enrichment score (NES) ≥ 1 or ≤ − 1, nominal *P* < 0.05, and false discovery rate (FDR) < 0.25. Pathway topology was visualized via enrichment maps, highlighting interconnected biological modules.

### Data sources and instrumental variable selection

GWAS summary statistics for osteoporosis were obtained from 7,751 cases and 476,847 controls through the GWAS Catalog (accession code GCST90038656) [12]. eQTLs corresponding to DEGs were sourced from the eQTLGen Consortium (Phase 2, whole-blood cis-eQTLs) [13], while pQTLs were retrieved from the deCODE proteomics cohort (*N* = 35,559 Icelandic individuals) (https://www.decode.com/summarydata/) [14].

To satisfy the core assumptions of Mendelian randomization, instrumental variables (IVs) were selected through a multi-stage filtering protocol. First, SNPs with genome-wide significance (*P* < 5.0 × 10⁻⁸) were extracted from cis-eQTL data, followed by linkage disequilibrium (LD) clumping (*r*² < 0.1 within ± 10,000 kb genomic windows) using PLINK v1.9 to ensure genetic independence [15]. Horizontal pleiotropy was rigorously assessed through MR-Egger intercept tests [16], with variants showing evidence of pleiotropic effects (*P* < 0.05) removed from subsequent analyses. The strength of IVs was quantified using the F-statistic (computed as R^2^/K × (n − k − 1)/(1 − R^2^)), where R^2^ represents the proportion of exposure variance explained by IVs, n the eQTL sample size, and k the number of IVs [17]. To mitigate weak instrument bias, all SNPs with F -statistics < 10 were excluded, consistent with established thresholds for MR studies [18].

### Multi-omics integration analysis and Mendelian randomization analysis

Our study integrated RNA-seq, eQTL, and pQTL datasets within a MR framework to infer causal effects of transcriptomic and proteomic markers on osteoporosis. First, DEGs identified from RNA-seq data were intersected with cis-eQTLs from the eQTLGen Consortium and pQTLs from the deCODE proteomics study. These overlapping loci served as candidate molecular instruments for MR.

For statistical modeling, we primarily applied the inverse-variance weighted (IVW) method, as implemented in the TwoSampleMR R package (v0.5.7), to estimate the causal effects of gene or protein expression on osteoporosis risk [19]. To evaluate potential heterogeneity across instrumental variables, IVW estimates were compared with MR-Egger regression, which simultaneously assesses directional pleiotropy through intercept tests (Bonferroni-adjusted *P* > 0.05) [20]. Sensitivity analyses included Cochran’s Q statistic to quantify between-SNP heterogeneity (*P* < 0.01 threshold) and leave-one-out analyses to identify disproportionately influential variants, with causal estimate stability confirmed when effect size variations remained within 95% confidence intervals [21]. Potential pleiotropic bias was further interrogated through funnel plot asymmetry analysis, while significant associations (IVW *P* < 0.05) were visualized via ggplot2-generated scatter plots (exposure-outcome effect concordance) and forest plots (single-SNP effect consistency). All analyses were conducted in R v4.2.2 with analytical reproducibility ensured through version-controlled scripting.

This multi-omics integration strategy builds on recent methodological advances in combining transcriptomic and proteomic QTLs with GWAS summary statistics to improve drug target discovery [14]. Our analytic pipeline thus ensured that the identified candidate, CPXM1, was supported by concordant causal evidence across RNA, eQTL, and pQTL layers.

All scripts used in this study for data preprocessing, Mendelian randomization and visualization are openly available at: https://github.com/yangyoung724/Multiomics-MR-analysis. The repository includes detailed documentation and instructions for reproducing the analyses presented in this manuscript.

### RNA-seq validation of CPXM1 expression

Public RNA-seq data from osteoporosis patients and controls (GSE35958) [22] were reanalyzed to validate CPXM1 expression differences. Specifically, mesenchymal stem cells (MSCs) were isolated from femoral head bone marrow of two distinct cohorts: (1) elderly osteoporosis patients (*n* = 5) suffered from osteoporosis were isolated from femoral heads after low-energy fracture of the femoral neck, and (2) age-matched non-osteoporotic donors (*n* = 4) undergoing total hip arthroplasty for osteoarthritis or developmental hip dysplasia. Raw expression data underwent log2 transformation to stabilize variance across intensity ranges, followed by quantile normalization using the normalize.quantiles function from the R package *preprocessCore* (v1.56.0) to eliminate inter-sample technical variability. Differential expression analysis was performed with DESeq2 (v1.38.3), defining significance as *P* < 0.05. CPXM1-specific expression patterns were visualized through customized volcano plots highlighting log2 fold changes against *P*-values.

### Quantitative real-time PCR analysis

Femoral bone samples were collected from 3-month-old and 18-month-old mice. Total RNA was isolated from cells using RNAiso Plus reagent (Takara, Cat# 9108) following the manufacturer’s protocols. RNA purity and concentration were quantified with a Nanovue spectrophotometer (GE Healthcare Life Sciences, Marlborough, MA, USA). cDNA synthesis was performed with the PrimeScript RT reagent kit (Takara, Cat# RR047A). Quantitative real-time PCR was conducted on the MxPro-Mx3000P system using SYBR Premix Ex Taq™ (Takara, Cat# RR820A), with each sample analyzed in triplicate.

Gene expression levels were normalized to GAPDH as an internal control, and relative quantification was calculated using the 2 ^− △ △^Ct method. Primer sequences were as follows: CPXM1-F, 5’-CACTGTCTATGCTGGCACGA-3’, CPXM1-R, 5’- GGCTCCATTGATGACGTTGC3’; GAPDH-F, 5’- AACTCCCACTCTTCCACCTTC-3’, GAPDH-R, 5’- CCTGTTGCTGTAGCCGTATTC-3’.

### Tissue-specific expression profiling

The expression profile of CPXM1 across human tissues was characterized using RNA-seq data from the Genotype-Tissue Expression (GTEx) database (v8) [23], encompassing 54 normal tissue types. Protein-level validation was performed through the Human Protein Atlas (HPA) [24]. This conserved expression pattern across transcriptional and translational layers underscores CPXM1’s potential involvement in osteoporosis pathogenesis.

### Phenome-wide association analysis

To comprehensively assess potential horizontal pleiotropy and off-target effects of CPXM1, we conducted a phenome-wide association study (PheWAS) using the AstraZeneca PheWAS Portal (https://azphewas.com/) [25]. This platform leverages exome sequencing data from approximately 450,000 participants in the UK Biobank, encompassing ~ 15,500 binary (e.g., disease diagnoses) and ~ 1,500 continuous (e.g., biomarker levels) phenotypes. Statistical associations between CPXM1 genetic instruments and phenotypes were evaluated genome-wide, with multiple testing correction applied using a stringent significance threshold of *P* < 2*10^− 9^, as recommended by the portal’s default parameters.

### Computational drug repurposing analysis

To identify pharmacological agents targeting CPXM1-associated pathways, we leveraged the Drug Signatures Database (DSigDB v1.0, http://dsigdb.tanlab.org/DSigDBv1.0/) [26], a comprehensive resource containing 22,527 experimentally validated drug-gene interaction profiles.

## Results

### Age-related trabecular deterioration revealed by histomorphometry

**Fig. 2:**
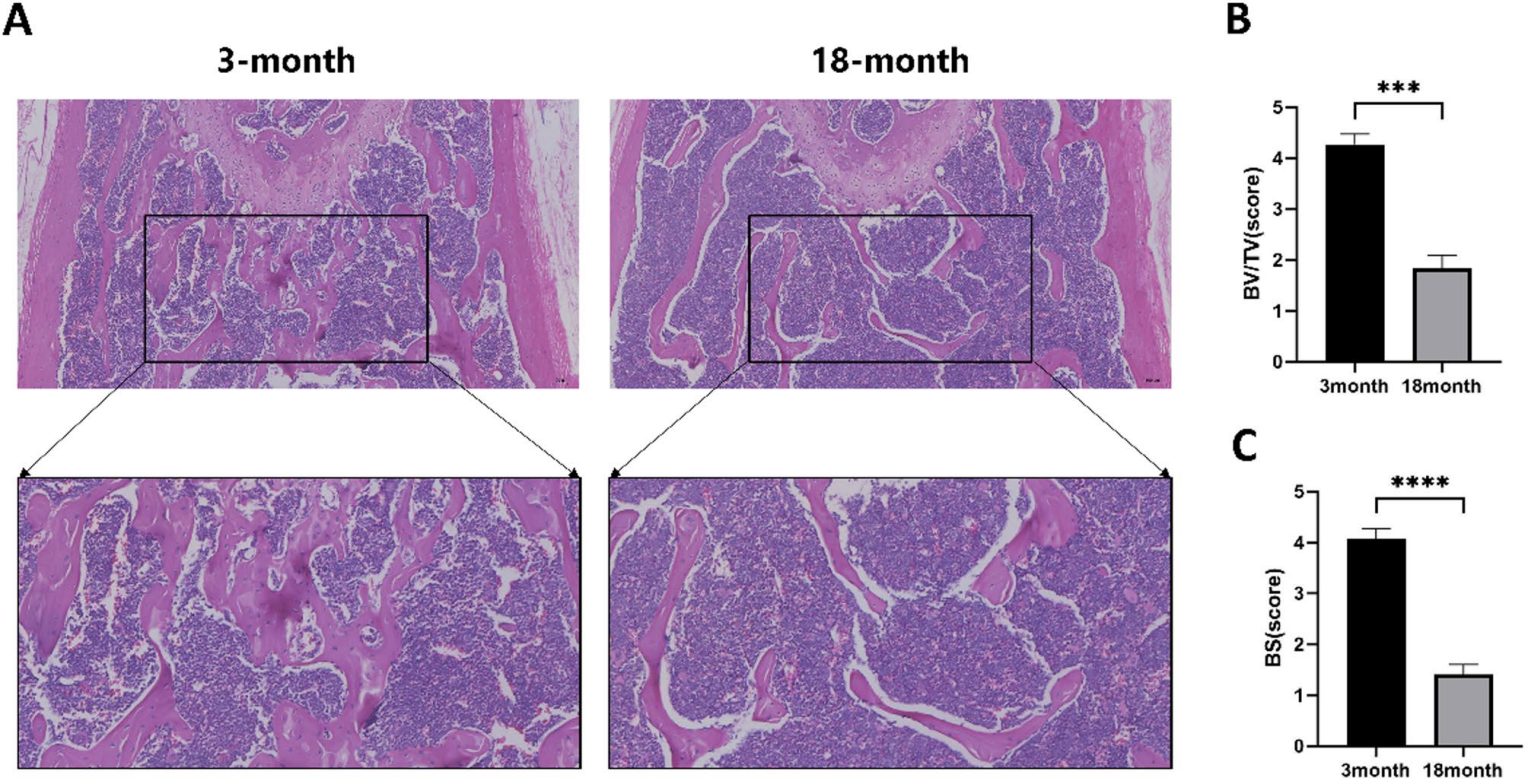
Age-related changes in bone microstructure. (**A**) Representative histological images of bone tissue sections from 3-month and 18-month-old animals. The top images show the overall bone structure, while the bottom panels are magnified views of the boxed areas. (**B**) Quantification of bone volume to total volume (BV/TV) at 3 and 18 months, with significantly reduced values in the 18-month group (*P* < 0.001). (**C**) Measurement of bone surface (BS) at 3 and 18 months, showing a marked decrease in the 18-month group (*P* < 0.0001). Statistical significance was determined by t-test

Histomorphometric analysis of hematoxylin and eosin (H&E)-stained sections revealed pronounced age-related deterioration in trabecular bone structure. Compared to 3-month-old mice, 18-month-old specimens exhibited a marked reduction in trabecular number and width, along with irregular and disrupted trabecular surfaces. The trabeculae appeared sparse and slender, and the marrow cavity was notably expanded (Fig. 2A).

In addition, there was a significant increase in both the number and size of bone marrow adipocytes in the aged group. Quantitative analysis of bone structural parameters based on H&E sections further confirmed these morphological observations: both bone volume fraction (BV/TV) and bone surface area (BS) were significantly decreased in 18-month-old mice (Fig. 2B-C). These findings indicate substantial bone loss and trabecular degradation associated with aging.

### Transcriptomic profiling of age-associated osteoporosis changes

Raw RNA-seq reads underwent rigorous quality filtering to remove adapter sequences and low-quality bases (Phred score < 20) using Trimmomatic v0.39. Reads containing > 5 ambiguous bases (N) were excluded, yielding 6 high-confidence libraries (18-month-old: O-18 m-1/2/3; 3-month-old: Y-3 m-1/2/3) with an average of 42.6 million clean reads per sample (Table S1). Alignment to the Mus musculus reference genome (ENSEMBL GRCm39, May 2021 release).

**Fig. 3:**
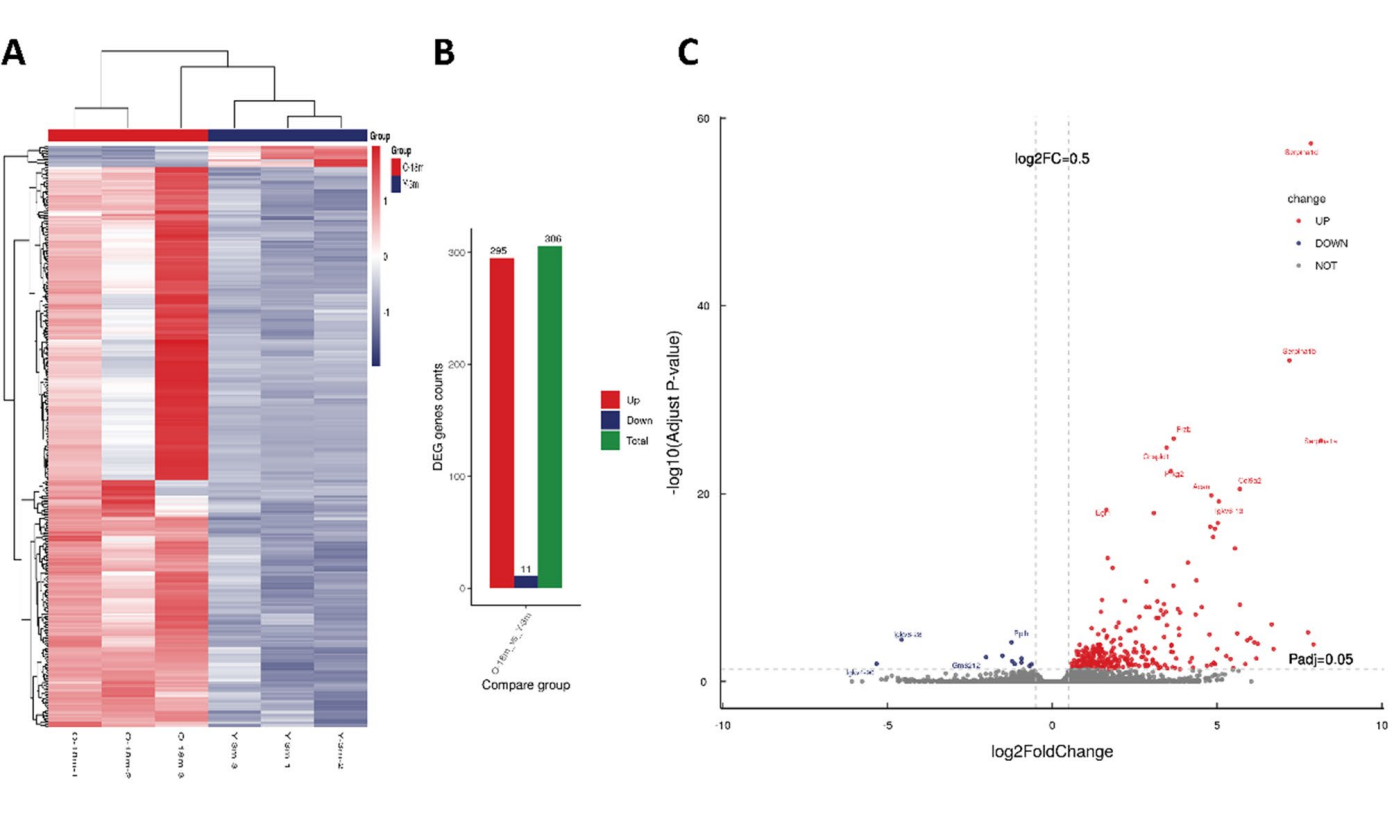
Transcriptomic profiling of age-associated osteoporosis. (**A**) Heatmap of differentially expressed genes (DEGs) between 3-month-old and 18-month-old mice. Gene expression data were normalized using z-scores. Red indicates upregulation, while blue indicates downregulation of gene expression. (**B**) Bar plot showing the number of upregulated, downregulated, and total DEGs identified from RNA-seq analysis. A total of 306 DEGs were identified, with 295 genes upregulated and 11 downregulated. (**C**) Volcano plot illustrating the significance of DEGs with respect to fold change and adjusted *p*-values. Upregulated genes are shown in red, downregulated genes in blue, and non-significant genes in gray. The vertical dashed line represents a log2 fold change threshold of ± 0.5, and the horizontal dashed line represents the adjusted *P* -value threshold of 0.05

Clustered heatmaps of the up-regulated and down-regulated DEGs expression were shown in Figs. 3A, based on z-scores normalization, DESeq2 analysis of read count data identified 306 differentially expressed genes (DEGs, |log2FC| >0.5, adjusted *P* < 0.05): 295 genes were upregulated (e.g., *Serpina1d*, log2FC = 7.8485, adjusted *P* = 4.8355E-58) and 11 downregulated (e.g., *Igkv8-28*, log2FC = -4.573, adjusted *P* = 0.000035668) (Fig. 3B-C) (Table S2).

### GSEA reveals age-associated imbalance in bone homeostasis

**Fig. 4:**
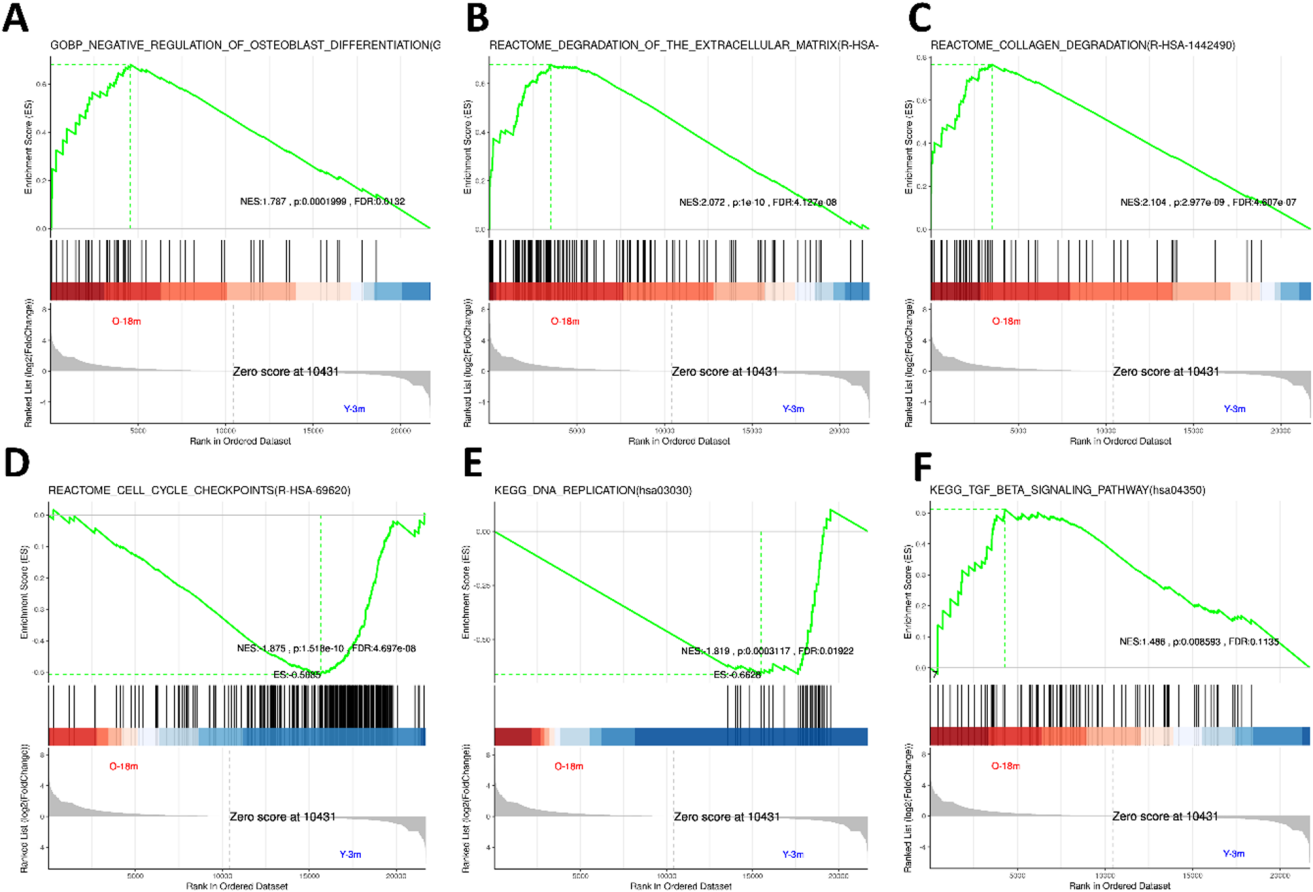
The enrichment of DEGs in 18-month-old versus 3-month-old mice was analyzed via gene set enrichment analysis (GSEA). (**A**) Negative Regulation of Osteoblast Differentiation. (**B**) Degradation of the Extracellular Matrix. (**C**) Collagen Degradation. (**D**) Cell Cycle Checkpoints. (**E**) DNA Replication. (**F**) TGF-β signaling pathway. The green dashed line illustrates the trajectory of the Enrichment Score (ES) for the gene set, with the Y-axis quantifying the ES magnitude. Individual genes within the analyzed pathway or Gene Ontology (GO) term are denoted by black vertical ticks along the ranked gene list. The accompanying heatmap visualizes expression-level changes across all genes, encoded by signal-to-noise ratio (SNR) or log2 fold change values. Red hues indicate upregulated expression (values > 0), while blue hues represent downregulation (values < 0), with color intensity scaled to reflect the absolute magnitude of differential expression

GSEA uncovered significant dysregulation of bone homeostasis pathways in 18-month-old versus 3-month-old mice. Pathways driving bone resorption were robustly activated in aged bone, including Degradation of the Extracellular Matrix (NES = 2.072, *P* = 1 × 10⁻¹⁰, FDR = 4.13 × 10⁻⁸) (Fig. 4B) and Collagen Degradation (NES = 2.104, *P* = 2.98 × 10⁻⁹, FDR = 4.61 × 10⁻⁷) (Fig. 4C), reflecting elevated proteolytic activity linked to osteoclast hyperactivity. Concurrently, aged mice exhibited marked suppression of bone formation, evidenced by enrichment of Negative Regulation of Osteoblast Differentiation (NES = 1.787, *P* = 0.0002, FDR = 0.013) (Fig. 4A). Cell cycle regulation was severely compromised in aged osteoprogenitors, with significant downregulation of Cell Cycle Checkpoints (NES=-1.875, *P* = 1.52 × 10⁻¹⁰, FDR = 4.70 × 10⁻⁸) (Fig. 4D) and DNA Replication pathway activation (NES=-1.819, *P* = 0.0003, FDR = 0.019) (Fig. 4E). While the TGF-β signaling pathway exhibited enrichment in aged bone (NES = 1.486, nominal *P* = 0.0086, FDR = 0.114) (Fig. 4F).

### Mendelian randomization between eQTLs and osteoporosis

**Fig. 5:**
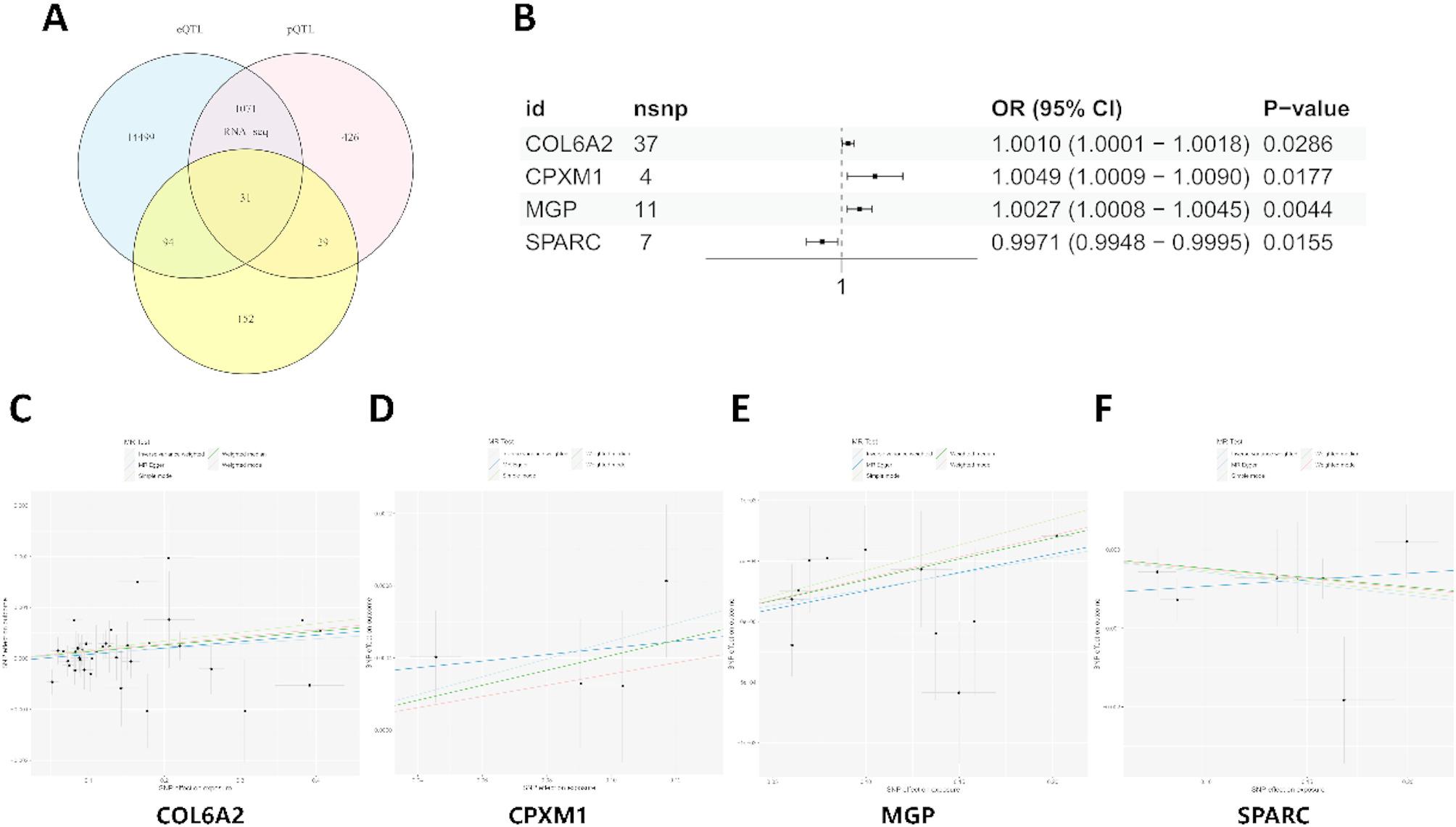
Mendelian randomization analysis of significant eQTLs. (**A**) Venn diagram of three datasets. (**B**) Forest plot of the associations between genetically determined 4 gene expression with the risk of osteoporosis. Abbreviations: CI, confidence interval; OR, odds ratio; SNP, single nucleotide polymorphism. (**C**) Scatter plots illustrating the MR estimates for the significant causality between COL6A2 and the risk of osteoporosis. (**D**) Scatter plots illustrating the MR estimates for the significant causality between CPXM1 and the risk of osteoporosis. (**E**) Scatter plots illustrating the MR estimates for the significant causality between MGP and the risk of osteoporosis. (**F**) Scatter plots illustrating the MR estimates for the significant causality between SPARC and the risk of osteoporosis. Positive associations between gene expression and osteoporosis risk were represented by upward-sloping regression lines (left to right), suggesting a potential promoting influence. Conversely, downward-sloping lines denoted negative associations, implying protective effects of gene expression. Horizontal and vertical error bars delineate the 95% confidence intervals for each correlation estimate

Intersection of RNA-seq-derived DEGs with cis-eQTL and pQTL datasets identified 31 candidate genes exhibiting transcriptional and post-translational regulatory signatures (Fig. 5A). MR (IVW method) using these genes as exposures against the GCST90038656 osteoporosis GWAS cohort revealed four genes with significant causal associations (*P* < 0.05). Three genes conferred increased osteoporosis risk: Collagen type VI alpha 2 chain (COL6A2) (OR = 1.001, 95% confidence interval (CI) 1.0001–1.0018, *P* = 0.029) (Fig. 5B-C), Carboxypeptidase X, M14 family member 1 (CPXM1) (OR = 1.005, 95% CI 1.001–1.009, *P* = 0.018) (Fig. 5B, D), and Matrix Gla protein (MGP) (OR = 1.003, 95% CI 1.001–1.005, *P* = 0.004) (Fig. 5B, E). Conversely, Secreted protein acidic and cysteine rich (SPARC) (OR = 0.997, 95% CI 0.995–0.999, *P* = 0.016) (Fig. 5B, F) exhibited protective effects.

### Mendelian randomization between pQTLs and osteoporosis

**Fig. 6:**
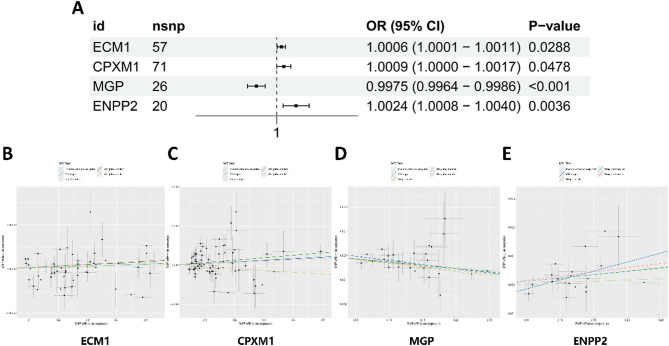
Mendelian randomization analysis of significant pQTLs. (**A**) Forest plot of pQTL MR associations with osteoporosis risk. Abbreviations: CI, confidence interval; OR, odds ratio; SNP, single nucleotide polymorphism. (**B**) ECM1 in pQTL-MR analysis. (**C**) CPXM1 in pQTL-MR analysis. (**D**) MGP in pQTL-MR analysis. (**E**) ENPP2 in pQTL-MR analysis. The scatter plot illustrates the genetic association effects between SNP-exposure (x-axis) and SNP-outcome relationships (y-axis), with vertical and horizontal error bars indicating 95% confidence intervals. Regression lines represent causal effect estimates derived from five Mendelian randomization (MR) methods: inverse-variance weighted (IVW), MR-Egger, weighted median, simple mode, and weighted mode. Slopes of these lines reflect the magnitude and direction of putative causal relationships. Abbreviations: MR, Mendelian randomization; SNP, single-nucleotide polymorphism

Further MR analysis using pQTLs of the 31 overlapping genes identified four protein-level causal associations with osteoporosis risk (IVW method, *P* < 0.05). Extracellular matrix protein 1 (ECM1) (OR = 1.0006, 95% CI 1.0001–1.0011, *P* = 0.029) (Fig. 6A-B), CPXM1 (OR = 1.0009, 95% CI 1.0000–1.0017, *P* = 0.048) (Fig. 6A, C), and Ectonucleotide pyrophosphatase/phosphodiesterase 2 (ENPP2) (OR = 1.002, 95% CI 1.001–1.004, *P* = 0.004) (Fig. 6A, E). In contrast, MGP showed a protective effect (OR = 0.997, 95% CI 0.996–0.999, *P* = 7.26 × 10⁻⁶), opposite to its eqtl results (Fig. 6A, D).

### Validation of CPXM1 in osteoporosis

**Fig. 7:**
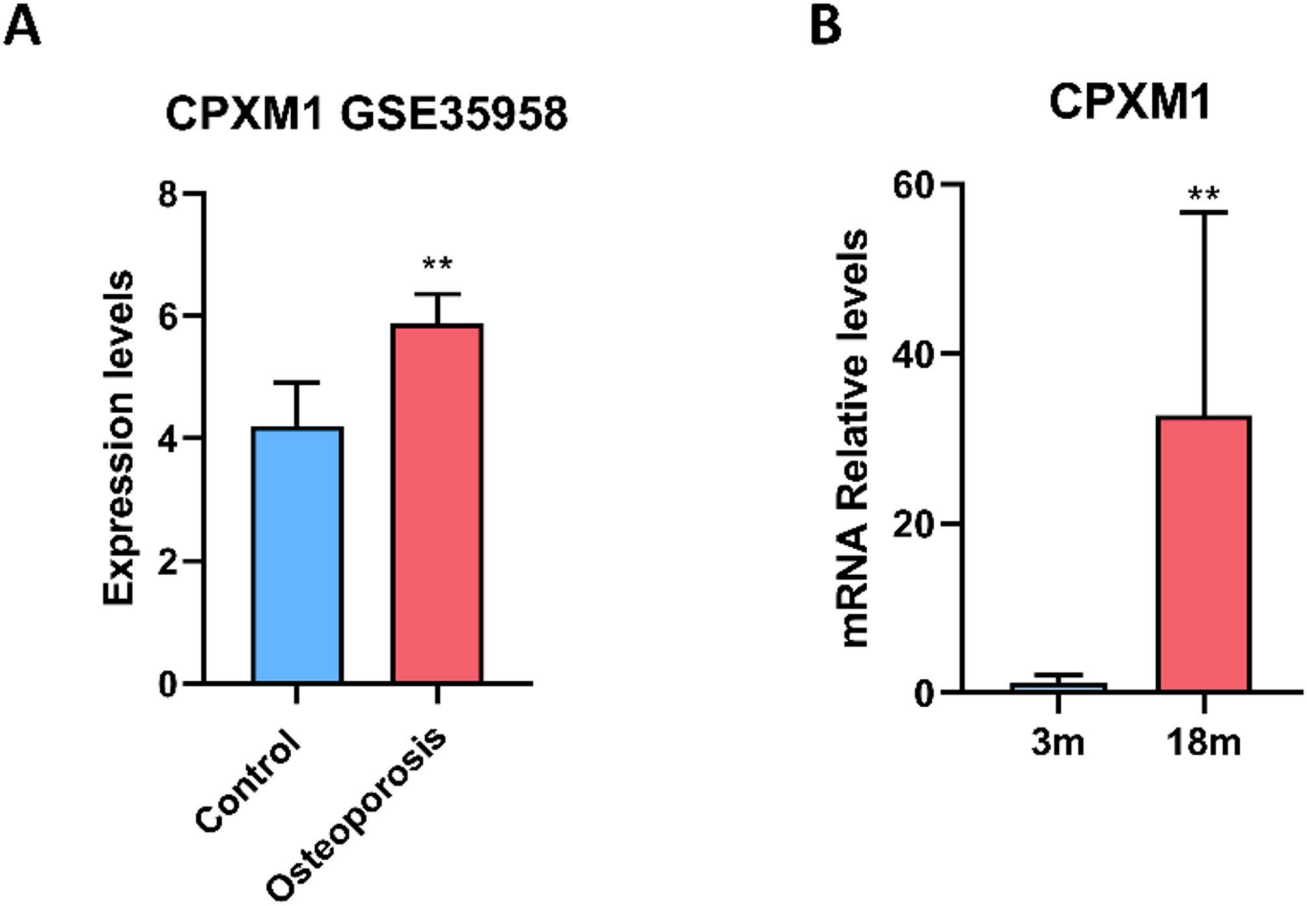
Validation of CPXM1 upregulation in osteoporosis. (**A**) CPXM1 expression in mesenchymal stem cells (MSCs) from osteoporosis patients (GSE35958). (**B**) CPXM1 expression in murine femoral bone was validated by qRT-PCR analysis of tissues from 3-month-old (young adult) and 18-month-old (aged) mice. ^**^P < 0.01

To validate the role of CPXM1 in osteoporosis, we analyzed RNA-seq data from MSCs isolated from femoral head bone marrow of elderly osteoporosis patients and age-matched non-osteoporotic controls (GSE35958). After log2 transformation and quantile normalization, differential expression analysis using DESeq2 revealed significant upregulation of CPXM1 in osteoporosis patients (Fig. 7A) (Table S3).

To further confirm this finding in an aging model, femoral bone tissues from 3-month-old and 18-month-old mice were analyzed by qRT-PCR. CPXM1 expression was markedly elevated in aged mice compared to young (27-fold increase, *P* = 0.0035) (Fig. 7B) (Table S4), aligning with human RNA-seq results. Together, these multi-species datasets robustly demonstrated that CPXM1 expression was upregulated in osteoporotic conditions, supporting its potential role as a biomarker or therapeutic target in bone loss pathologies.

### CPXM1 expression in human tissues

**Fig. 8:**
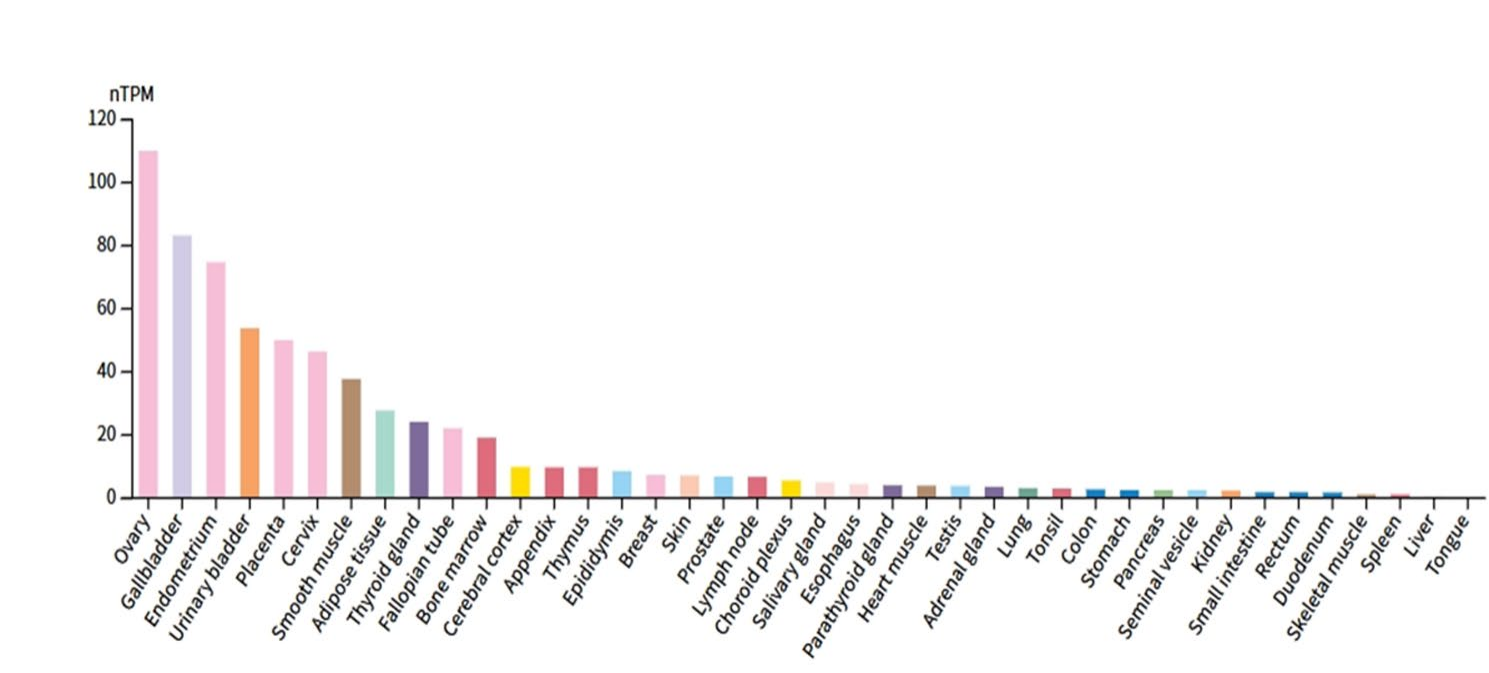
The expression of CPXM1 in human tissues

The tissue-specific expression profile of CPXM1 was analyzed using the Human Protein Atlas. Results demonstrated that CPXM1 is predominantly expressed in the ovary, gallbladder, and endometrium (Fig. 8). This tissue-specific pattern may provide an explanation for the target organs and pathways through which CPXM1 exerts its biological effects.

### PheWAS portal analysis

**Fig. 9:**
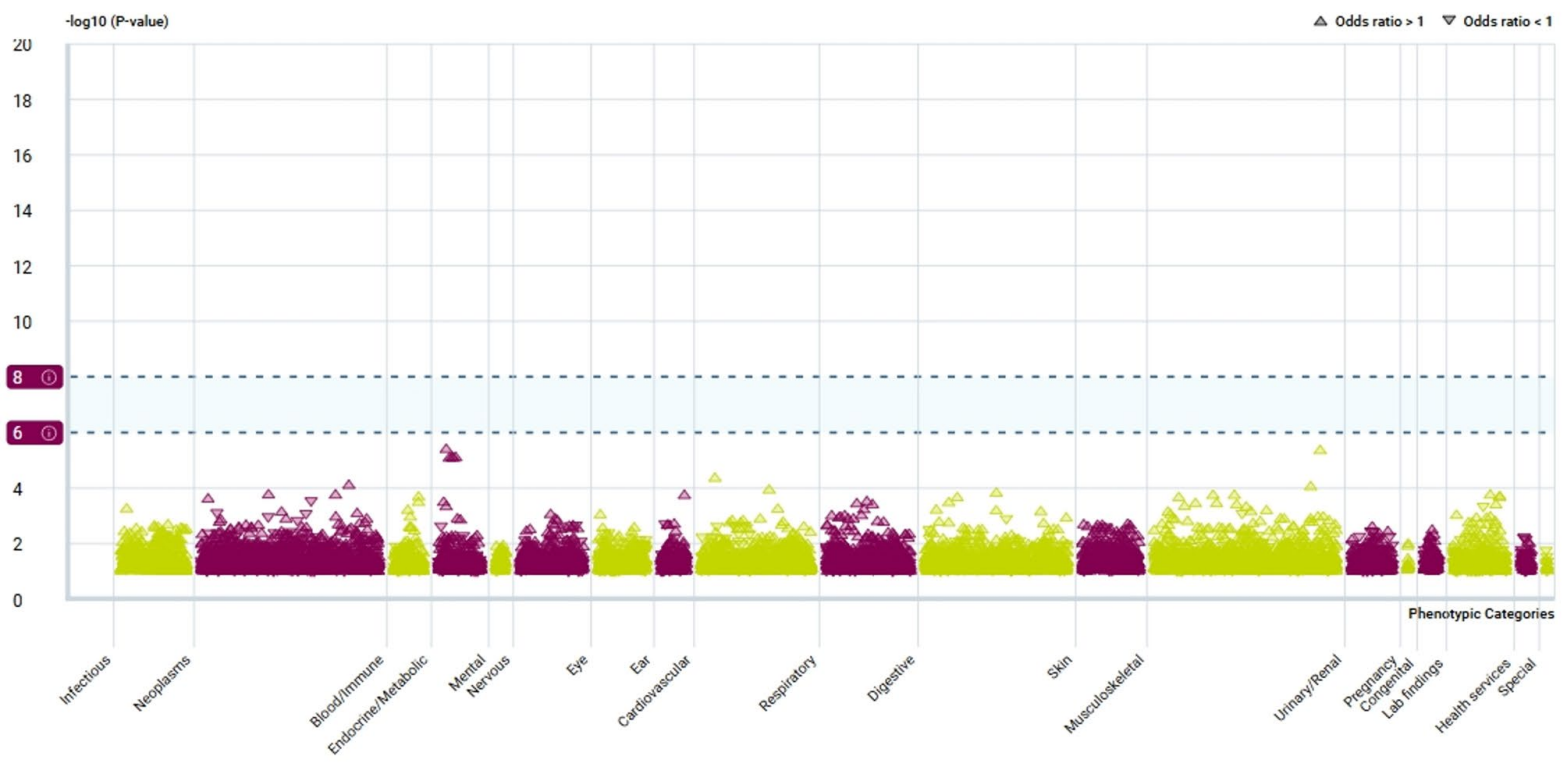
CPXM1 PheWAS associations with binary phenotypes

The PheWAS analysis revealed no genome-wide significant associations between CPXM1 and other phenotypes in the PheWAS Portal (*P* < 5 × 10⁻⁸) (Fig. 9). These findings corroborate the validity of our results and indicate minimal risk of adverse drug reactions or unintended horizontal pleiotropic effects associated with therapeutic targeting of CPXM1.

### Candidate drug prediction for CPXM1

In our investigation using the DSigDB drug database, we identified promising drug candidates that may target CPXM1 for osteoporosis treatment (Table 1). Notably, Doxorubicin (CTD 00005874) showed a significant association with a *P*-value of 0.0375, an adjusted *P*-value of 0.0833, and an extraordinarily high odds ratio of 19,250.00, indicating strong potential for therapeutic efficacy. Similarly, 5-Fluorouracil (CTD 00005987) displayed a *P*-value of 0.0601 and an odds ratio of 18,798.00, and 2-Methylcholine (CTD 00002006) had a *P*-value of 0.0833 and an odds ratio of 18,334.00, indicating relevance despite not achieving traditional significance. Collectively, these findings highlight the viability of Doxorubicin, 5-Fluorouracil, and 2-Methylcholine as therapeutic candidates targeting CPXM1 in osteoporosis, warranting further preclinical and clinical investigations to assess their efficacy and safety in this context.

**Table 1:** Drug candidates were discovered using the DSigDB database

Name	*P*-value	Adjusted *P* -value	Odds Ratio	Combined score
doxorubicin CTD 00005874	0.0375	0.0833	19250.00	63205.83
5-Fluorouracil CTD 00005987	0.0601	0.0833	18798.00	52855.26
2-Methylcholine CTD 00002006	0.0833	0.0833	18334.00	45565.67

## Discussion

Our multi-omics MR framework, integrating RNA-seq, eQTL, and pQTL data, identified CPXM1 as a novel causal mediator of osteoporosis risk. Through transcriptomic and proteomic validation in human and murine models, we demonstrated that CPXM1 upregulation is robustly associated with age-related trabecular bone loss and osteoporotic phenotypes. The concordant risk effects observed across both eQTL and pQTL analyses (*P* < 0.05), coupled with the absence of genome-wide pleiotropy in PheWAS (*P* < 5 × 10⁻⁸), underscore CPXM1 as a prioritized therapeutic target for osteoporosis.

Comprehensive histomorphometric and transcriptomic analyses have elucidated significant age-associated deterioration in bone microarchitecture and homeostatic regulation. Histological evaluations revealed that 18-month-old mice exhibited pronounced structural degradation of trabecular bone, characterized by reduced trabecular number and thickness, increased marrow adiposity, and disrupted trabecular surfaces. These morphological changes were emblematic of senile osteoporosis and align with previous findings on age-induced skeletal fragility [27]. Quantitative reductions in BV/TV and BS further confirmed the loss of bone mass.

Parallel transcriptomic profiling identified 306 DEGs, with a predominant upregulation pattern, indicating extensive transcriptional reprogramming in aged bone tissue. GSEA revealed that these DEGs were enriched in pathways associated with extracellular matrix degradation, collagen breakdown, and suppression of osteoblast differentiation, reflecting a shift towards catabolic bone remodeling [28]. Furthermore, the downregulation of cell cycle checkpoint and DNA replication pathways suggests impaired proliferation of osteoprogenitor cells, contributing to diminished bone formation capacity [29]. Notably, a modest enrichment of the TGF-β signaling pathway was observed, potentially indicating a compensatory response or involvement in dysregulated remodeling processes [30]. Collectively, these findings highlight a multifaceted molecular basis for age-related bone loss, involving both enhanced osteoclastic activity and compromised osteoblastic renewal.

MR analysis provided novel insights into the causal roles of specific genes and proteins in osteoporosis development. By integrating cis-eQTL, pQTL, and RNA-seq data, four candidate genes were identified with significant causal effects on osteoporosis risk, including COL6A2, CPXM1, MGP, and SPARC. COL6A2 and CPXM1, both associated with extracellular matrix composition and remodeling, were found to increase susceptibility to osteoporosis [31], whereas SPARC exhibited a protective effect, consistent with its known roles in bone matrix mineralization and osteoblast function [32]. Further pQTL-based MR analysis also identified ECM1 and ENPP2 as risk-promoting factors, highlighting the contribution of extracellular matrix organization and lipid metabolism to osteoporosis pathophysiology [33]. Interestingly, MGP showed opposing effects at the transcript and protein levels, suggesting complex regulatory mechanisms potentially involving post-transcriptional modifications or protein activity regulation [34]. CPXM1, in particular, was validated through independent datasets, demonstrating robust upregulation in bone marrow-derived mesenchymal stem cells from osteoporotic patients as well as in aged murine bone tissue, suggesting that CPXM1 may serve as a potential biomarker or therapeutic target for age-related bone loss [22].

CPXM1 is a secreted protein known for its collagen-binding properties [35]. Although research on CPXM1’s direct involvement in bone metabolism, particularly in osteoporosis, is still limited, its role in bone matrix remodeling is of increasing interest. CPXM1’s interaction with collagen, suggested it could influence the structural integrity of the bone matrix. Given that osteoporosis was characterized by reduced bone mineral density and increased bone fragility, often associated with abnormal bone matrix degradation and remodeling, CPXM1 could play a significant role in regulating bone strength and density by modulating these processes. Moreover, Kim et al. suggested that CPXM1 acted as a positive regulator of adipogenesis, providing indirect evidence of its involvement in cell differentiation, which may be relevant to bone cell differentiation as well [36]. These findings support the hypothesis that CPXM1 might be involved in bone matrix remodeling, affecting bone density and strength, and consequently playing a role in the pathophysiology of osteoporosis. Overall, while more research is required, CPXM1’s potential role in bone metabolism and osteoporosis is a promising area for future investigation, as it could lead to new therapeutic targets for osteoporosis treatment.

The expression analysis of CPXM1 revealed a tissue-specific profile, with high expression levels in the ovary, gallbladder, and endometrium, suggesting that CPXM1 may play a role in the biological functions of these organs. Furthermore, the PheWAS analysis did not show any significant associations between CPXM1 and other phenotypes, indicating a low risk of adverse drug reactions or unintended effects from therapeutic targeting of CPXM1. In the drug prediction analysis, several promising candidates for osteoporosis treatment were identified, including Doxorubicin, 5-Fluorouracil, and 2-Methylcholine. These drugs showed strong associations with CPXM1, particularly Doxorubicin, which demonstrated an exceptionally high odds ratio, suggesting its potential as a therapeutic option. These findings provide a foundation for further research into CPXM1-targeting therapies for osteoporosis.

While this study provides novel insights into the molecular mechanisms of age-related osteoporosis, several limitations must be acknowledged. First, caution is warranted in extrapolating findings from mouse models to humans. The reliance on murine models, though invaluable for studying trabecular microarchitecture, may not fully recapitulate human bone remodeling dynamics due to species-specific differences in osteoclast-osteoblast coupling and hormonal regulation [37]. Species-specific differences in bone metabolism and osteocyte lacunar density have been well documented, which may confound direct translation of results from animal studies to human pathology [38, 39]. Future work integrating human genetic and transcriptomic resources, as well as patient-derived bone tissues, will be essential to strengthen translational relevance. Second, the exclusive use of European-ancestry GWAS and eQTL/pQTL datasets limits the generalizability of findings to ethnically diverse populations, as genetic architecture and allele frequencies of osteoporosis-associated loci vary across ancestries. Third, although anticancer drugs such as doxorubicin and 5-fluorouracil are highlighted by our computational repurposing screen, their strong cytotoxic and senescence-inducing properties limit their feasibility as osteoporosis treatments. Doxorubicin has been reported to drive therapy-induced senescence and bone loss [40], while 5-fluorouracil reduced osteoblast proliferation and bone formation [41, 42]. Thus, these compounds should not be considered therapeutic candidates; rather, they may serve as mechanistic probes to interrogate stress- and senescence-related pathways in bone remodeling. Moreover, recent studies suggest that cholinergic signaling may exert protective effects on bone by modulating osteoblast and osteoclast activity [43, 44]. This highlights a potentially underexplored neuroskeletal regulatory axis that warrants further discussion in the context of drug repurposing for osteoporosis. Fourth, while our study utilized bulk RNA-seq datasets, the availability of single-cell RNA-seq (scRNA-seq) from human bone marrow and bone tissues provides opportunities for higher-resolution dissection of disease-related cellular subpopulations [45, 46]. Future integration of scRNA-seq with eQTL and pQTL datasets could help refine causal inference and improve target prioritization. Finally, the role of CPXM1 requires further clarification. Although CPXM1 is categorized within the M14 metallocarboxypeptidase family, both database annotations and sequence analyses indicate it lacks key residues necessary for enzymatic activity. Functional characterization of the murine homolog CPX-1 further supports this, showing that the protein is secreted, contains a discoidin-like domain, and binds collagen via this domain, while exhibiting no catalytic activity [35]. These findings suggest that CPXM1 may serve as an ECM-interacting scaffold, potentially involved in extracellular matrix organization or signal modulation, rather than functioning as a classical proteolytic enzyme. Further functional validation, including protein-level assays (e.g., ELISA or antibody-based assays), is needed to confirm CPXM1’s role in bone biology. Future studies should focus on investigating its interaction with ECM components, the kinetics of these interactions, and the impact of specific mutations in the discoidin-like domain to better understand its precise function in bone matrix remodeling and signal modulation. Similarly, interpretation of MGP expression requires caution, as it has been reported to promote bone formation through Wnt/β-catenin activation [47], yet al.so to inhibit osteoclast differentiation and bone resorption under different conditions [48]. Such context-dependent effects emphasize the complexity of bone remodeling pathways and the need for multi-layered validation. Taken together, these considerations underscore the importance of distinguishing mechanistic probes from therapeutic candidates, exercising caution in cross-species generalization, and incorporating emerging high-resolution omics resources. While CPXM1 emerges as a promising candidate, its biological function should be further investigated in the context of ECM remodeling and osteoblast regulation, supported by both in vitro and in vivo validation.

In conclusion, our study presents CPXM1 as a promising drug target for osteoporosis, leveraging a multi-omics MR approach to uncover novel molecular insights. The integration of genetic, transcriptomic, and proteomic data provides a robust platform for drug discovery in osteoporosis, with implications for future therapeutic strategies. Further validation and clinical trials will be critical in assessing the efficacy and safety of targeting CPXM1 in osteoporosis treatment.

## Supplementary Information

Below is the link to the electronic supplementary material.

Supplementary Material 1

Supplementary Material 2

Supplementary Material 3

Supplementary Material 4

## Data Availability

We have deposited the raw sequencing data in the Sequence Read Archive (SRA) under accession number PRJNA 1305700.
